# Author Correction: LIM domain-wide comprehensive virtual mutagenesis provides structural rationale for cardiomyopathy mutations in CSRP3

**DOI:** 10.1038/s41598-022-08526-0

**Published:** 2022-03-14

**Authors:** Pankaj Kumar Chauhan, Ramanathan Sowdhamini

**Affiliations:** National Centre for Biological Sciences (Tata Institute of Fundamental Research), GKVK Campus, Bangalore, Karnataka 560065 India

Correction to: *Scientific Reports* 10.1038/s41598-022-07553-1, published online 3 March 2022

The original version of this Article contained an error in the spelling of the author Ramanathan Sowdhamini which was incorrectly given as Sowdhamini Ramanathan.

The original Article has been corrected.

