# Supplementary material for: LIM domain-wide comprehensive virtual mutagenesis provides structural rationale for cardiomyopathy mutations in CSRP3
Source: Sci Rep. 2022 Mar 3;12:3562. doi: 10.1038/s41598-022-07553-1 (PMC8894373; doi:10.1038/s41598-022-07553-1)
Supplement: Supplementary file 1 — Supplementary Information. [file 41598_2022_7553_MOESM1_ESM.pdf]

Supporting Information

LIM domain-wide comprehensive virtual mutagenesis provides structural rationale for cardiomyopathy mutations in CSRP3

Pankaj Kumar Chauhan and R. Sowdhamini

National Centre for Biological Sciences (Tata Institute of Fundamental Research), GKVK Campus, Bangalore Karnataka 560065 INDIA

Supplementary Material and Methods

Stability analysis energy calculations

The stability analysis was carried out using FoldX [1]. It performs a free energy calculation using an empirical force-field and outputs the Gibbs free energy of folding (kcal/mol). This energy is composed of the different terms as described in the equation (i). The Van der Waals terms (ΔG_{vdw}), hydrophobic (ΔG_{solvH}), polar (ΔG_{solvP}), a persistent water interaction (ΔG_{wb}), hydrogen bonds (ΔG_{hbond}), electrostatic (ΔG_{el}), an entropic penalty for fixing the backbone ($T\Delta S_{mc}$), entropy cost of fixing a side chain ($T\Delta S_{sc}$) and steric overlaps between atoms (ΔG_{clash}) are the individual components of Gibbs free energy. The pH, ionic strength and temperature used in analysis were kept at 7, 0.05 M and 298 K. Change in energy due to mutation ($\Delta\Delta G$) is calculated by subtracting $energy\ of\ mutant$ (ΔG_{mutant}) from $wild-type$ (ΔG_{WT}) as formulated in equation (ii).

$$\Delta G = \Delta G_{vdw} + \Delta G_{solvH} + \Delta G_{solvP} + \Delta G_{wb} + \Delta G_{hbond} + \Delta G_{el} + T\Delta S_{mc} + T\Delta S_{sc} + \Delta G_{clash}$$

(i)

$\Delta\Delta G = \Delta G_{mutant} - \Delta G_{WT}$ (ii)

FoldX first identifies residues having bad torsion angles, or Vander Waals' clashes, or total energy and corrects them using RepairPDB command. Here it carries out small optimization of side chains to eliminate small Vander Waals' clashes. Further, it mutates residues with bad energy and their neighbours to themselves to explore different rotamer combinations so that new energy minima can be achieved.

BuildModel is the main command in the stability analysis. It generates modelled structures of mutant protein. This command also ensures that the same neighbours are moved in the WT and in the mutant protein leading to generation of two PDBs (mutant and its corresponding WT).

Solvent accessible surface area

FreeSASA, an open-source tool was utilised for inferring total and polar solvent accessible area change due to each substitution in mutational landscape [2]. For this, high-precision calculation parameter (1000 slides per atom) was set using Lee and Richards (L&R) algorithm [3]. LIM1 and LIM2 mutations' total SASA were plotted against mutational landscape.

Contact Map Analysis

Contact map analysis of the MD trajectories can be helpful to acknowledge the lifetime of contacts across the structure or new conformational states traversed by a system. CONAN MAP [4] was utilised for this task. Only protein, excluding the TIP3P atoms, was considered from the trajectories in this analysis, and inter-residue contacts as well as correlation were calculated. All the frames of the MD trajectories were used for the study with an interval of 1 ns.

Inter Residue Contact Analysis

CONAN uses three cut-offs for inter-residue distance calculation:

r_{cut}: This is the primary cut-off value. Any residue pair without any atoms within this cut-off is disregarded.

r_{inter}: This is the cut-off value under which interactions are formed.

r_{inter}^{high} This is the cut-off value over which interactions are broken.

The inter-residue distance is defined as:

A main cut-off r_{cut}=1 nm, and interactions defined using the same cut-off, r_{inter}= r_{inter}^{high} =0.5 nm.

SI Figures

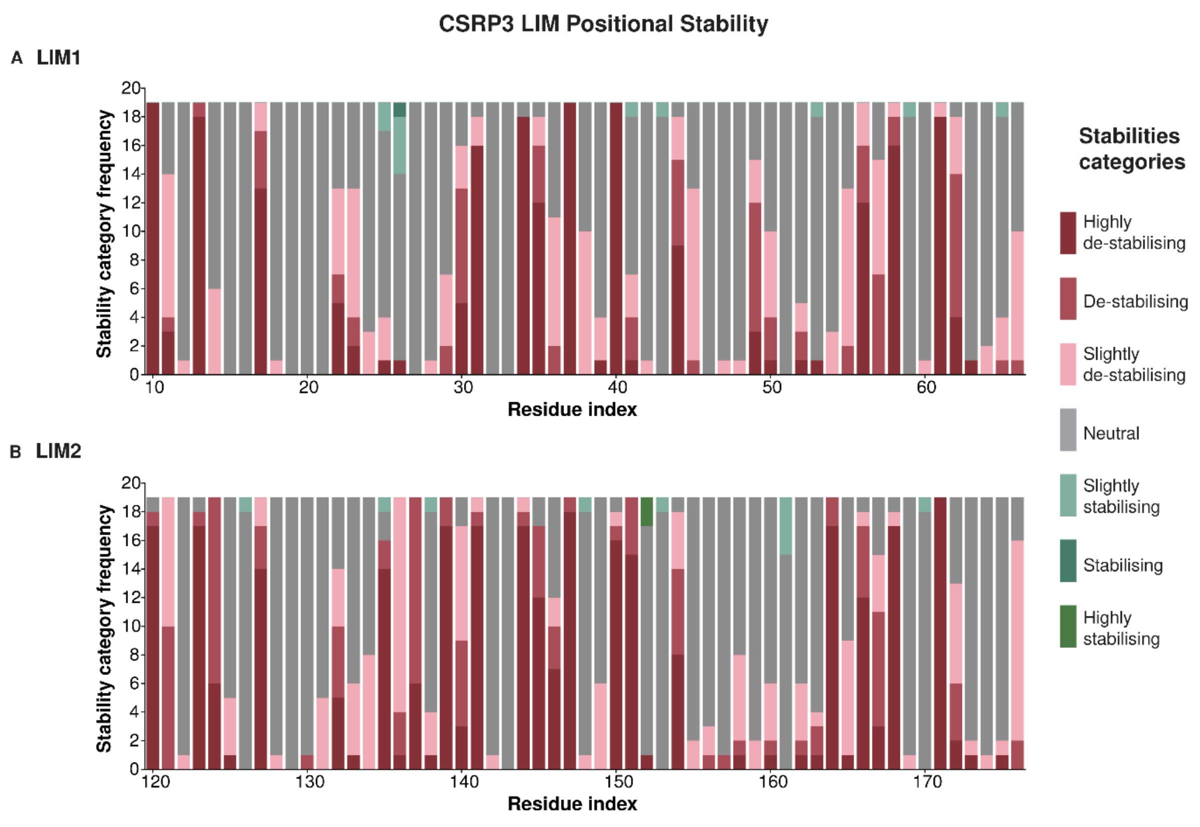

Figure S1: Histogram plots showing the mutational tolerance of each position in LIM1 and LIM2 domains of CSRP3. Severity of mutational effect is categorised into seven groups based on ΔΔG values as mentioned in the materials and methods. A) Frequency of different stability terms at each position in LIM1 domain. B) Frequency of different stability terms at each position in LIM2 domain.

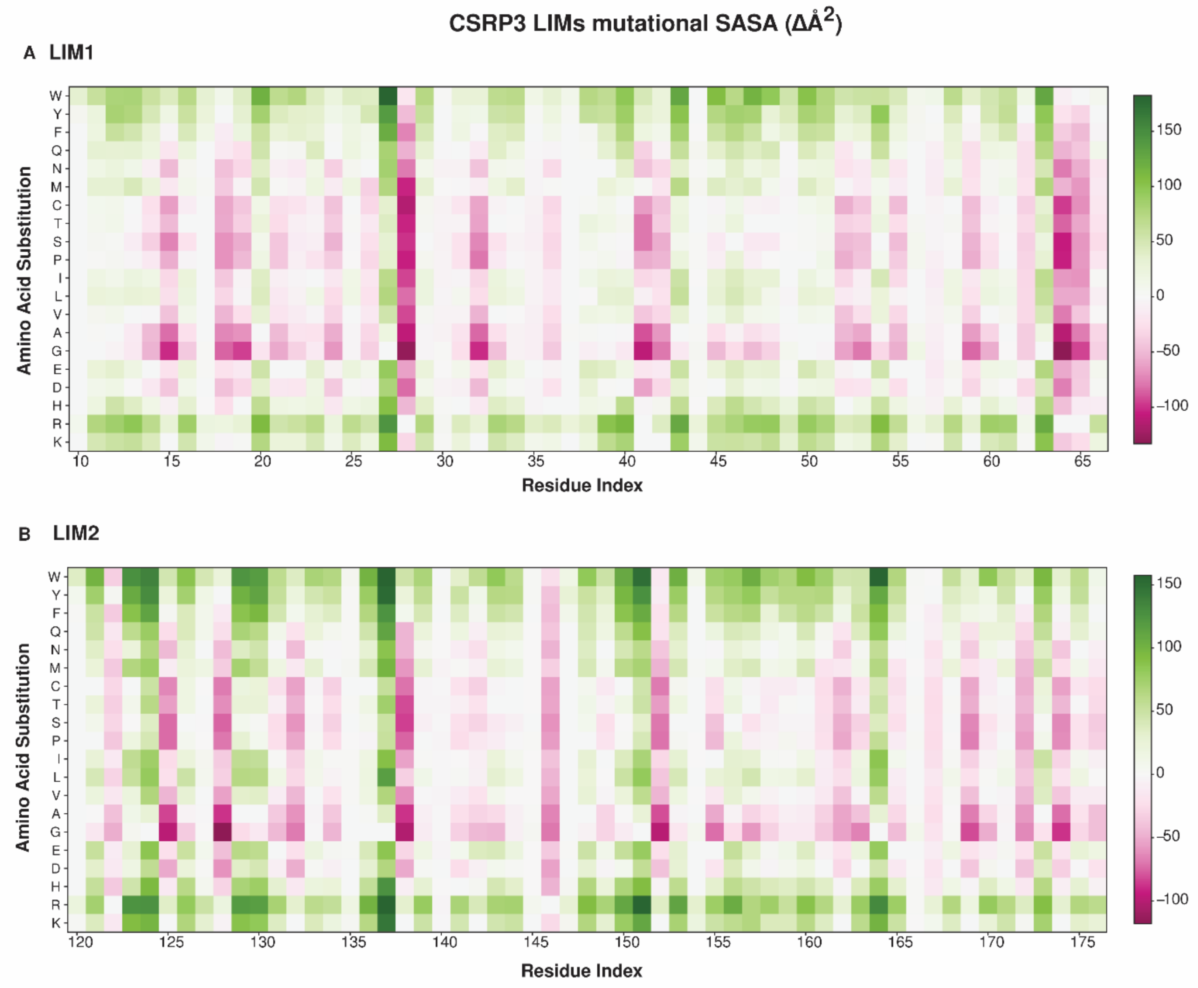

Figure S2: Heatmap of solvent accessible surface area (SASA) for LIM1 and LIM2 domains of CSRP3. Reported change in local SASA due to amino acid substitution at each position (ΔÅ²) is colored in shades of green (decreased in mutant compared to WT) and pink (increased in mutant compared to WT). A) LIM1 domain SASA heatmap and B) LIM2 domain SASA heatmap.

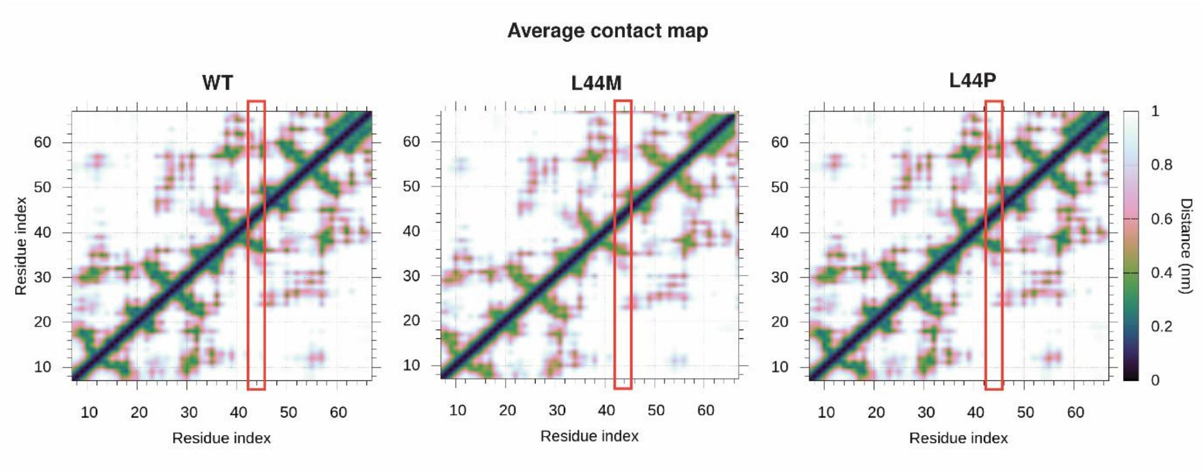

Figure S3: Contact map between residues pairs of LIM1 domain. CONAN tool default criterion was used for WT, L44M and L44P trajectories. Distance between residue pairs vary from 0-1 nm (dark blue to white color as seen in the legend). Red outlined box indicates L44 region.

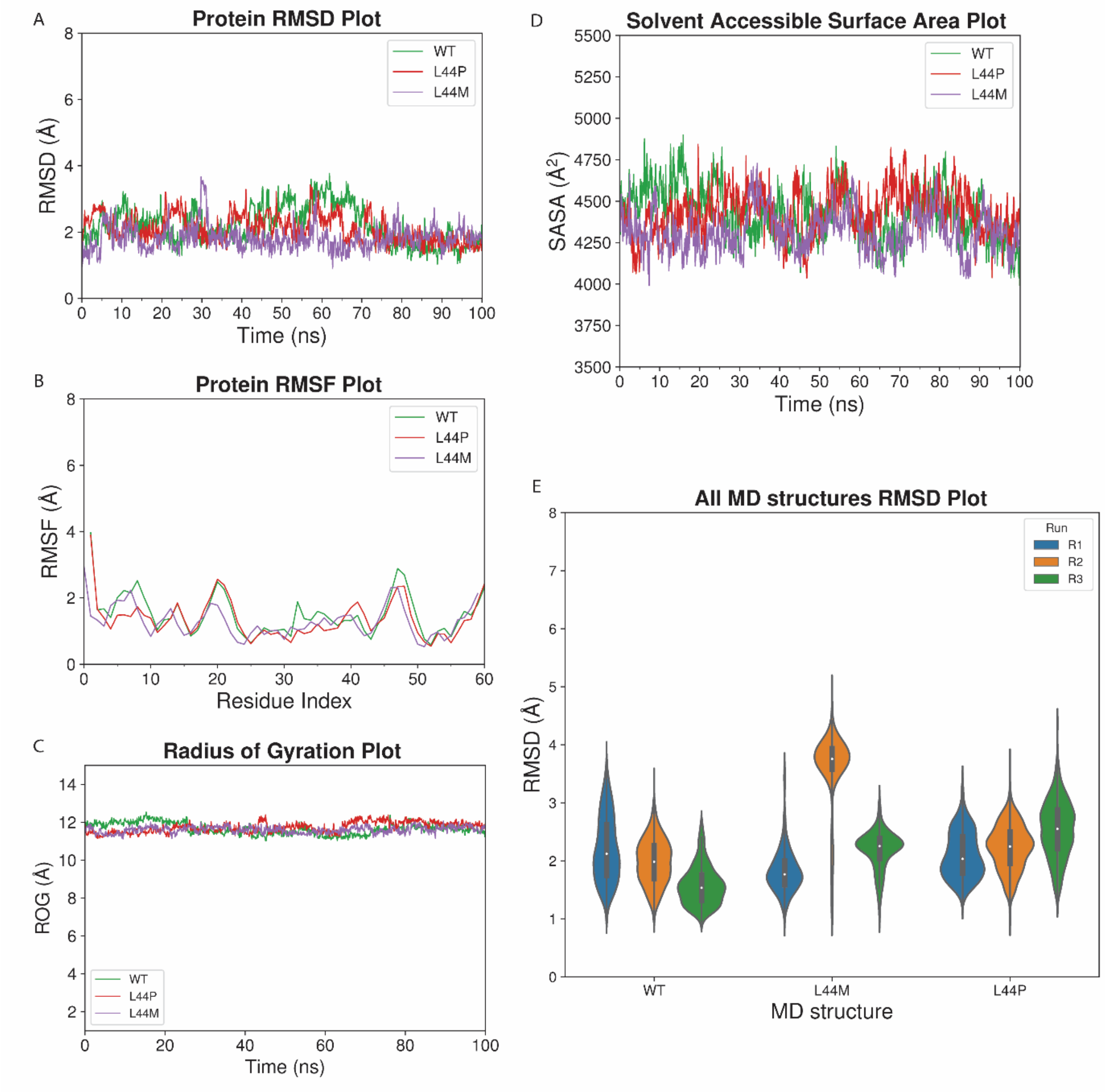

Figure S4: Simulation event analysis of LIM1 domain. Desmond package was used for simulation event analysis of WT,L44M and L44P trajectories labelled as green (WT), indigo (L44M) and red (L44P). A) Root mean square distance (RMSD) plot of the mentioned trajectories. B) Root mean square fluctuations (RMSF) of WT, L44M and L44P. C) Radius of Gyration (ROG) plot showing compactness of WT, L44M and L44P trajectories. D) Solvent accessible surface area (SASA) plot of the aforesaid trajectories. E) A violin plot showing distribution of RMSD values in the replicates of WT, L44M and L44P.

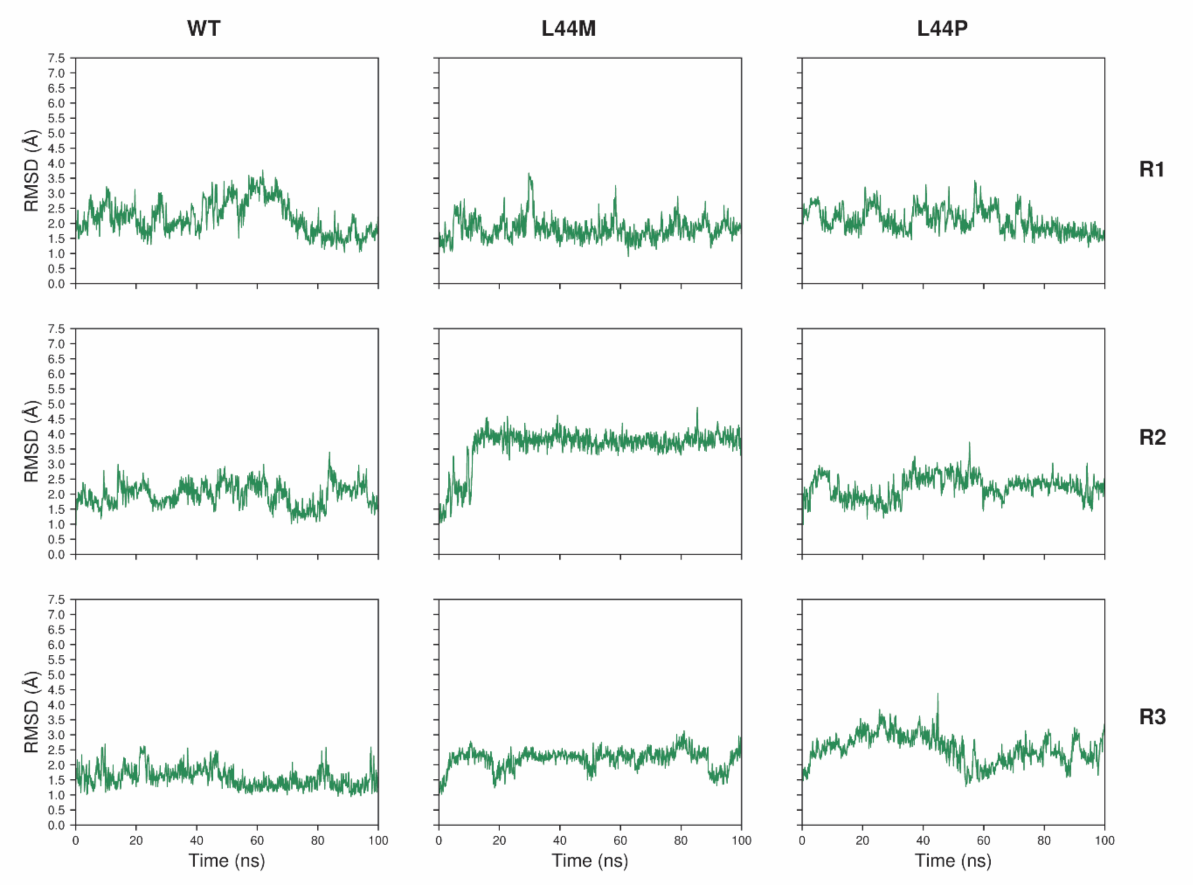

Figure S5: Root mean square distance (RMSD) plot in the replicates of WT, L44M and L44P trajectories. R1, R2 and R3 refer to RUN 1, RUN2 and RUN3 of MD simulation.

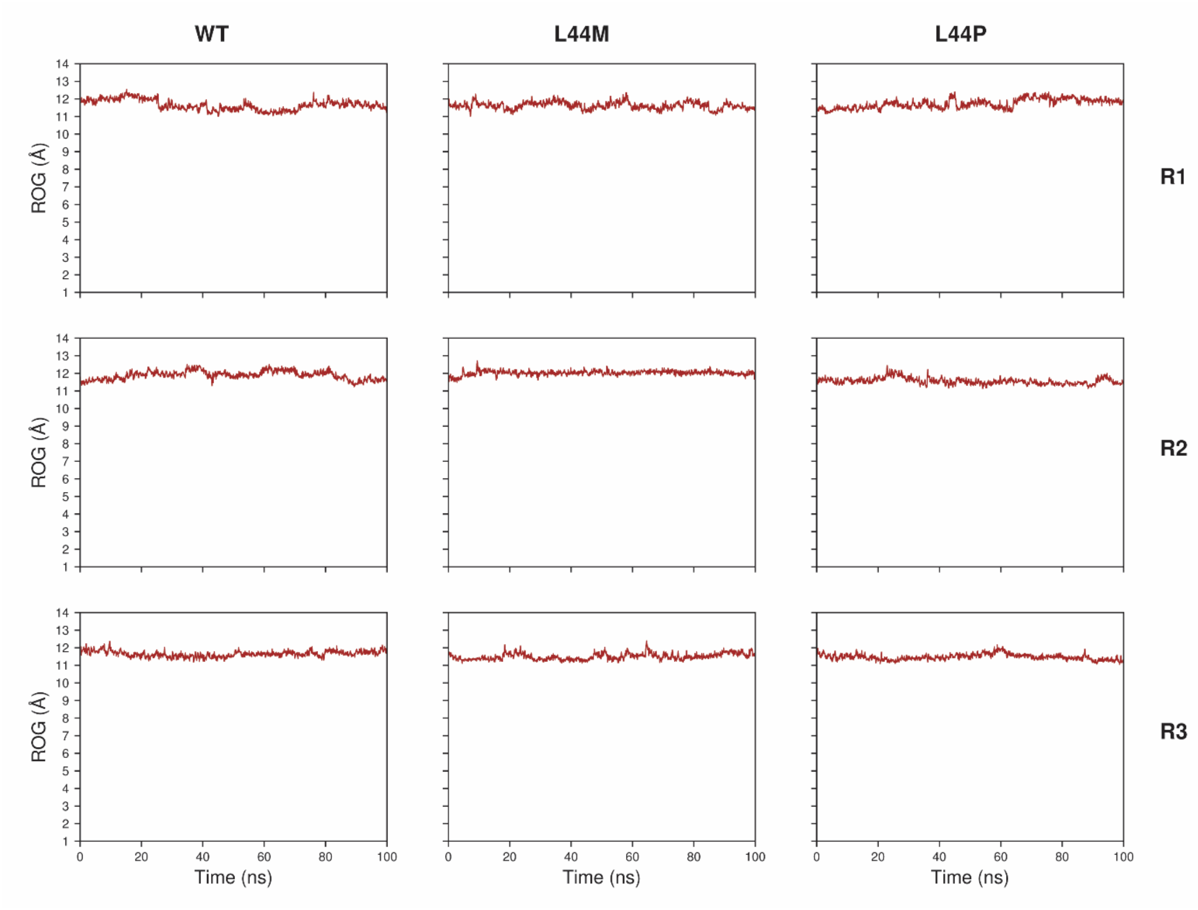

Figure S6: Radius of Gyration (ROG) plot in the replicates of WT, L44M and L44P trajectories. R1, R2 and R3 refer to RUN 1, RUN2 and RUN3 of MD simulation.

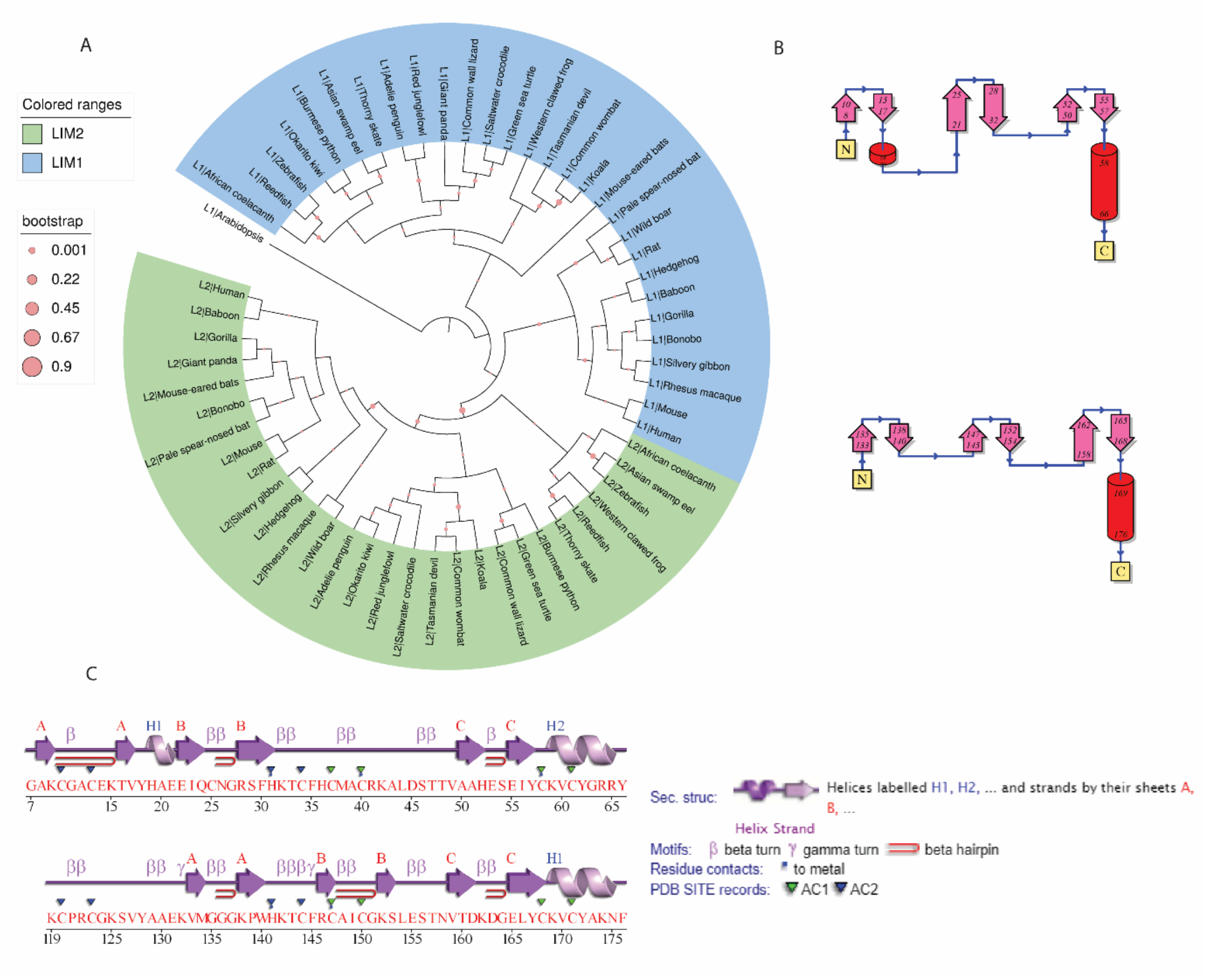

Figure S7: Sequence and structural differences between LIM1 and LIM2 domain of CSRP3. A) Maximum Likelihood Phylogeny of LIM1 and LIM2 in the representative eukaryotes with 1000 bootstrap iterations (pink circles) LIM and LIM2 are colored in blue and green respectively. B) Secondary structure connectivity map of LIM1 and LIM2 domains as seen in the pictorial database PDBsum . C) Two dimensional view of LIM domains of CSRP3 derived from PDBsum.

SI Tables

S. No.	AA position native substitution	Mean ΔΔG (kcal/mol)
1	C10C	0.00115387±0.00146757
2	G11G	1.27898e-14±2.12689e-14
3	K15K	-0.0209776±0.0211645
4	T16T	-0.0173856±0.0192991
5	H31H	-0.76799±0.467944
6	T33T	-0.0175463±0.018094
7	C34C	-0.00732078±0.00863648

8	L44L	-0.015029±0.0128481
9	S46S	-0.000206798±0.000314174
10	A51A	-2.13163e-15±7.8963e-15
11	C58C	-0.0375605±0.0310757
12	R64R	0.0210829±0.046674

Table S1: A table showing mean and standard deviation values of ΔΔG during self-substitution of native amino acid at the representative amino acid positions. These positions were either highly destabilising or neutral in our mutational landscape.

	**Mean RMSD (Å)**		
	**WT**	**L44M**	**L44P**
**RUN 1**	2.201±0.559	1.818±0.372	2.100±0.402
**RUN 2**	1.984±0.390	3.607±0.628	2.229±0.390
**RUN 3**	1.573±0.332	2.190±0.326	2.538±0.488

Table S2: Summary table showing the mean RMSD values for each replicate of WT, L44M and L44P trajectories.

**Organism**	**Common name**	**Taxa**
Xenopus tropicalis	Western clawed frog	Amphibian
Gallus gallus	Red junglefowl	Aves
Pygoscelis adeliae	Adelie penguin	Aves
Apteryx rowi	Okarito kiwi	Aves
Sus scrofa	Wild boar	Carnivore
Ailuropoda melanoleuca	Giant panda	Carnivore
Danio rerio	Zebrafish	Fish
Monopterus albus	Asian swamp eel	Fish
Erpetoichthys calabaricus	Reedfish	Fish
Amblyraja radiata	Thorny skate	Fish
Latimeria chalumnae	African coelacanth	Fish

Sarcophilus harrisii	Tasmanian devil	Marsupial
Phascolarctos cinereus	Koala	Marsupial
Vombatus ursinus	Common wombat	Marsupial
Podarcis muralis	Common wall lizard	Other vertebrate
Python bivittatus	Burmese python	Other vertebrate
Chelonia mydas	Green sea turtle	Other vertebrate
Crocodylus porosus	Saltwater crocodile	Other vertebrate
Myotis myotis	Mouse-eared bats	Placental
Phyllostomus discolor	Pale spear-nosed bat	Placental
Echinops telfairi	Hedgehog	Placental
*Arabidopsis thaliana**	arabidopsis	Plant
Homo sapiens	Human	Primate
Pan paniscus	Bonobo	Primate
Macaca mulatta	Rhesus macaque	Primate
Hylobates moloch	Silvery gibbon	Primate
Gorilla gorilla	Gorilla	Primate
Papio anubis	Baboon	Primate
Mus musculus	Mouse	Rodent
Rattus rattus	Rat	Rodent

*Table S3: Representative eukaryotes used for CSRP3 sequence conservation and phylogeny analysis. *Arabidopsis was used as out-group in the study.*

References

1. Guerois, R., Nielsen, J. E. & Serrano, L. Predicting changes in the stability of proteins and protein complexes: A study of more than 1000 mutations. *Journal of Molecular Biology* **320**, 369–387 (2002).

2. Mitternacht, S. FreeSASA: An open source C library for solvent accessible surface area calculations. *F1000Research* **5**, 189 (2016).

3. Lee, B. & Richards, F. M. The interpretation of protein structures: Estimation of static accessibility. *Journal of Molecular Biology* **55**, 379-IN4 (1971).

4. Mercadante, D., Gräter, F. & Daday, C. CONAN: A Tool to Decode Dynamical Information from Molecular Interaction Maps. *Biophysical Journal* **114**, 1267–1273 (2018).
